# Ultra-miniaturized HMSIW cavity-backed reconfigurable antenna diplexer employing dielectric fluids with wide frequency tuning range

**DOI:** 10.1038/s41598-024-79609-3

**Published:** 2024-11-13

**Authors:** Rusan Kumar Barik, Slawomir Koziel

**Affiliations:** 1 School of Microelectronics, Southern University of Science and Technology, Shenzhen, China; 2 Engineering Optimization and Modeling Center, Reykjavik University, Reykjavik, 102 Iceland; 3 Faculty of Electronics, Telecommunications and Informatics, Gdansk University of Technology, Gdansk, 80-233 Poland

**Keywords:** Engineering, Electrical and electronic engineering

## Abstract

This communication presents an ultra-miniaturized two-way frequency tunable antenna diplexer based on cavity-backed slots and dielectric fluids. The proposed antenna utilizes two half-mode substrate-integrated rectangular cavities loaded with slots and fluidic pockets. The conventional size reduction is achieved by employing half-mode cavities, whereas ultra-miniaturization is obtained by applying the slots, which provides additional capacitive loading. As the cavities are of unequal sizes, a weak cross-coupling path is created between the ports to obtain high isolation (> 30 dB). The isolation is further enhanced by loading the slots. Two mechanisms are analyzed to tune the frequency bands individually or simultaneously. Firstly, the width of the slots can be altered to tune the frequency bands. However, this method involves modification of the physical dimensions of the antenna. Secondly, fluidic vias are created on the bottom plane of the cavities. These can be filled with various dielectric liquids to achieve frequency reconfigurability without altering the physical dimensions of the antenna. To demonstrate the concepts considered, the prototype of the proposed antenna was fabricated and experimentally validated. The structure has a footprint of 0.045λ_g_^2^ and an isolation exceeding 33.4 dB. The operating frequencies are tunable in the range from 3.08 to 3.84 GHz (lower band) and from 4.97 to 6.33 GHz (upper band) by varying the dimensions of the slots whereas the operating frequencies are reconfigurable in the range from 2.74 to 3.38 GHz (lower band) and from 4.54 to 5.58 GHz (upper band), by employing microfluidic approach. As a result, the working frequencies may be varied in the range from 2.74 to 3.84 GHz (lower band) and from 4.54 to 6.33 GHz (upper band), making this antenna diplexer a competitive candidate for several communication systems. The cross-polarization levels, front-to-back ratio, and realized gain are greater than 19 dB, 18 dB, and 2. dBi, respectively. Excellent consistency is observed between full-wave simulation results and the measurement data.

## Introduction

The fast advancements in modern communication technologies necessitate the implementation of compact, cost-effective, high-performance, and multi-frequency antennas. The growing demand for dual-band single-fed antennas has drawn considerable attention from researchers due to their small size, excellent radiation properties, dual-frequency applications, and simple construction^1–3^. Nonetheless, the lack of band selection capability and inadequate isolation across the various operational bands limits the range of applicability of single-fed dual-frequency antennas. This issue may be alleviated by integrating high isolation diplexer circuits^4,5^ with the single-fed antenna to pick a certain frequency band. Adding a diplexer to an antenna increases the circuit’s complexity, which in turn raises its cost and enlarges the footprint. Researchers have thus devised dual-fed dual-band antennas with built-in diplexing capabilities to address the aforementioned needs.

Recently, several self-diplexing slot antennas (SDSA) have been designed based on microstrip and substrate-integrated waveguide (SIW) technologies^6–11^. In^6^, a self-diplexing planar inverted F antenna has been developed based on the microstrip technology. The substrate-integrated cavities loaded with a bowtie-ring slot^7^, U-shaped slot^8^, Y-shaped slot^9^, rectangular slots^10^, and arrow-shaped slots^11^. Although the antennas discussed above operate well, they are confined to the fixed frequency bands and have a large physical footprint.

In recent years, researchers have developed a number of reconfigurable dual-band antennas and self-diplexing antennas using semiconductor diodes and microfluidic channels^12–20^. Based on the varactor diodes, a dual-band reconfigurable eight-mode SIW antenna^12^ and dual-fed self-diplexing tunable antennas^13–15^ have been designed. Although these antennas exhibit excellent frequency tunability, their applicability is restricted due to the need for a biasing circuit, limited power-handling capabilities, and incompatibility with wearable or flexible electronic systems. However, many microfluidically reconfigurable dual-band antennas^16,17^ and self-multiplexing antennas^18–20^ have been created to address these problems. Dielectric liquid-based antennas provide excellent linearity, high power handling capacity, and are compatible with wearable electronics. These characteristics make them a promising option for dual-band systems.

In this brief, an ultra-miniaturized two-way frequency tunable antenna diplexer is presented. The proposed structure is embedded on two half-mode substrate-integrated cavities loaded with L-shaped slots and microfluidic channels. For verification, a prototype of the antenna has been manufactured and tested. The contributions of this work and the competitive features of our antenna are as follows:

1. The antenna diplexer covers a footprint area of 0.045λ_g_^2^, which is the most compact self-diplexing antenna in the literature to the best of the authors’ knowledge;
2. Two mechanisms for the frequency tunability are examined. The operating frequencies are tunable in the range from 4.05 to 4.56 GHz and from 6.19 to 7.26 GHz at lower and upper bands, respectively;
3. To validate the features, we construct equivalent circuits of the antenna diplexer with and without microfluidic channels;
4. The suggested antenna diplexer outperforms the reconfigurable self-diplexing designs available in the current literature in terms of isolation, which exceeds 33 dB.

## Methods

### Antenna diplexer design and analysis

**Fig. 1:**
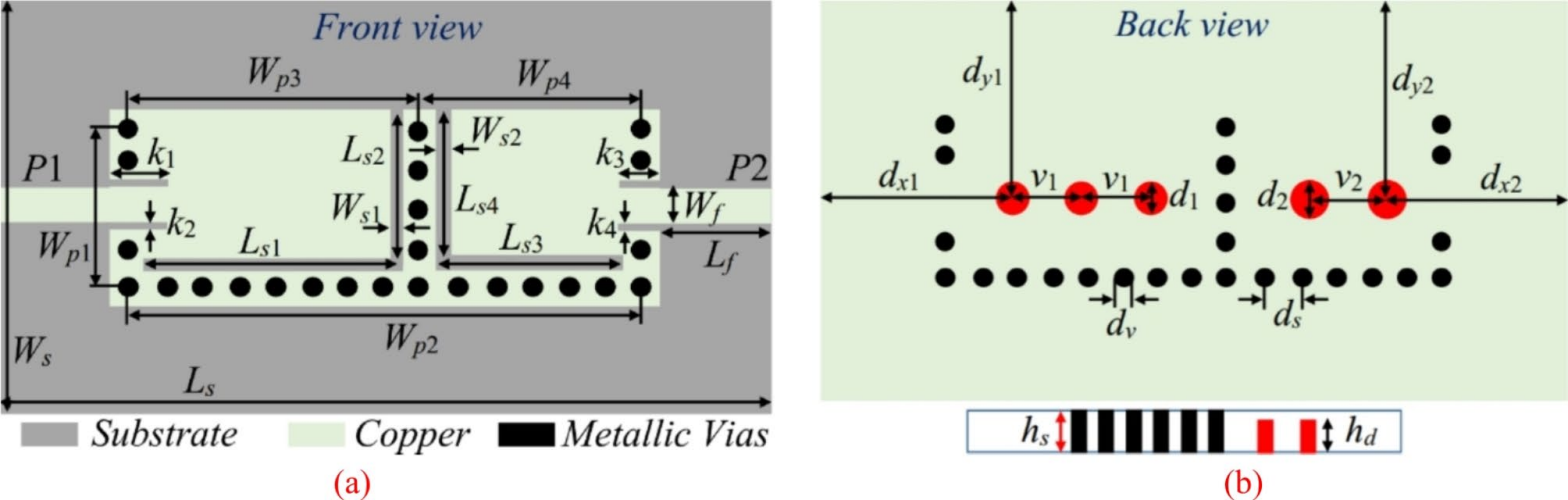
Schematic of the proposed tunable antenna diplexer. The final design parameter values are: *L*_*s*_ = 38, *W*_*s*_ = 20, *W*_*p*1_ = 8.4, *W*_*p*2_ = 26, *W*_*p*3_ = 16, *W*_*p*4_ = 10, *L*_*s*1_ = 14.4, *L*_*s*2_ = 7.9, *L*_*s*3_ = 8.4, *L*_*s*4_ = 7.7, *W*_*s*1_ = 0.5, *W*_*s*2_ = 0.7, *L*_*f*_ = 5.0, *W*_*f*_ = 2.16, *k*_1_ = 3.08, *k*_2_ = 0.211, *k*_3_ = 1.74, *k*_4_ = 0.211, *d*_*x*1_ = 10, *d*_*y*1_ = 10, *d*_*x*2_ = 8.5, *d*_*y*2_ = 10, *d*_1_ = 2.4, *v*_1_ = 4, *d*_2_ = 2.4, *v*_2_ = 4, *d*_*v*_ = 1.0, *d*_*s*_ = 2.0, *h*_*s*_ = 0.762, *h*_*d*_ = 0.562; unit: mm.

**Fig. 2:**
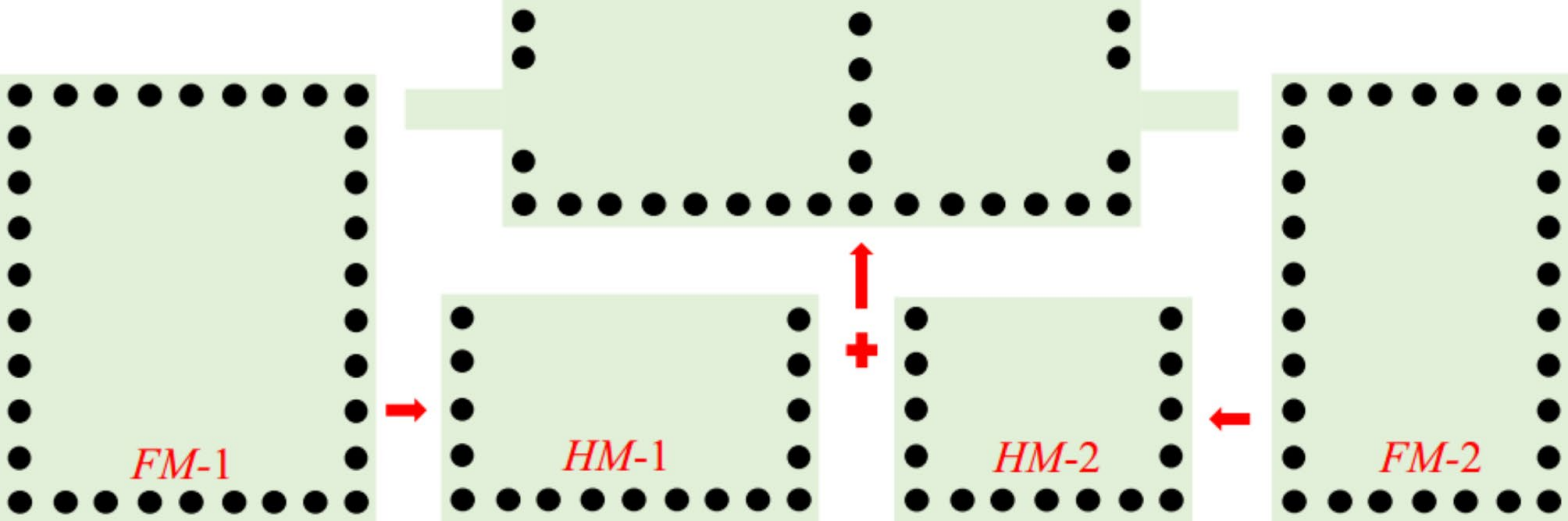
Conversion full-mode into half-mode cavities and creation of an antenna.

**Fig. 3:**
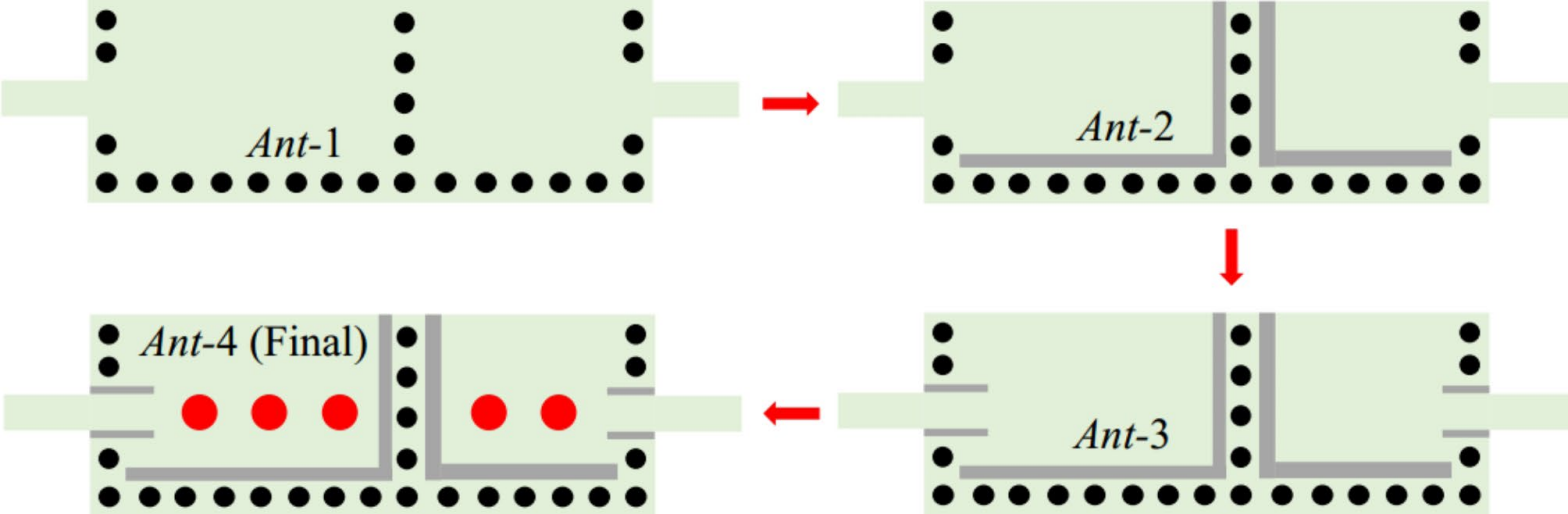
Step-by-step realization of the proposed tunable antenna diplexer.

**Fig. 4:**
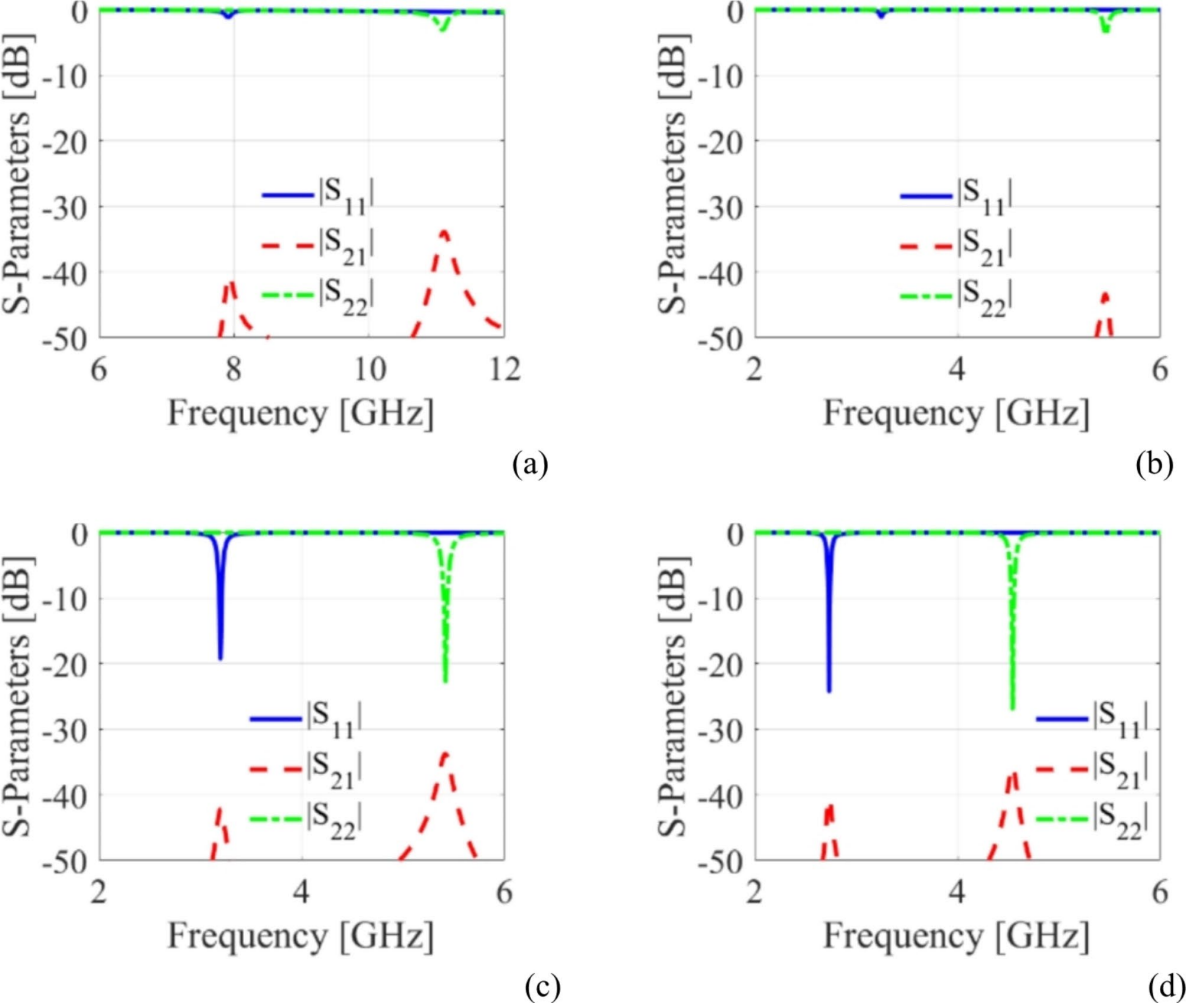
S-parameters of various antenna design stages: (**a**) Ant-1, (**b**) Ant-2, (**c**) Ant-3, and (**d**) Ant-4.

Figure 1 shows the final layout of the proposed reconfigurable antenna diplexer for sub-6 GHz applications. The suggested antenna diplexer is realized based on two half-mode (HM) substrate-integrated cavities loaded with L-shaped slots (LSS), microfluidic channels, and microstrip lines. The generation of HM cavities and step-by-step realization of the antenna diplexer are depicted in Figs. 2, 3, respectively. Initially, two HM cavities are formed by dividing two unequal full-mode cavities horizontally. The length, width, and fundamental mode frequencies of the HM cavities are computed as^5^:

1 $$\left\{ \begin{gathered} L_{{_{{eq}} }}^{{HM1/2}} = W_{{p3/4}} - 1.08\frac{{d_{v} ^{2} }}{{d_{s} }} + 0.1\frac{{d_{v} ^{2} }}{{W_{{p3/4}} }} \hfill \\ W_{{_{{eq}} }}^{{HM1/2}} = W_{{p1}} - 1.08\frac{{d_{v} ^{2} }}{{d_{s} }} + 0.1\frac{{d_{v} ^{2} }}{{W_{{p1}} }} \hfill \\ \end{gathered} \right.$$

2 $$f_{{110}}^{{HM1/2}} = \frac{1}{{2\sqrt {\mu \varepsilon } }}\sqrt {\left( {\frac{\pi }{{2W_{{_{{eq}} }}^{{HM1/2}} }}} \right)^{2} + \left( {\frac{\pi }{{L_{{_{{eq}} }}^{{HM1/2}} }}} \right)^{2} }$$

where *d*_*v*_, *d*_*s*_, *µ* = *µ*_0_*µ*_*r*_, and *ε* = *ε*_0_*ε*_*r*_ are the diameter, center-to-center spacing, permeability, and permittivity, respectively. The relationships of *d*_*v*_/*λ* ≤ 0.1 and *d*_*v*_/*d*_*s*_ ≥ 2 are applied to mitigate the energy leakage from the cavities^5^. Subsequently, two HM cavities are connected side-by-side and excited by two microstrip lines to create a conventional antenna (referred to as Ant-1) that operates at fundamental frequencies of 7.92 GHz and 11.2 GHz. The CST Microwave Studio software has been employed for the full-wave analysis. The EM-simulated *S*-parameters are shown in Fig. 4a. To realize sub-6 GHz frequency bands, two LSS are created on the top conductor, as shown in Ant-2. The corresponding *S*-parameters are depicted in Fig. 4b. It can be observed that the radiating frequencies are moved to lower values of 3.2 GHz and 5.42 GHz, which enables considerable miniaturization. Yet this version of the antenna achieves poor impedance matching at both ports. Subsequently, inline feeding is utilized at both ports to achieve the necessary impedance matching, as seen in Ant-3. The EM-simulated *S*-parameters of this antenna have been shown in Fig. 4c. It can be noted that the impedance matching is significantly improved at a level greater than − 19 dB. Finally, empty microfluidic pockets associated with HM cavities are generated at the bottom conductor (cf. Ant-4) and filled with various dielectric fluids to enable frequency reconfigurability. The diameter and height of the fluidic pockets are expressed by *d*_1_ = *d*_2_ = 2.4 mm and *h*_*d*_ = 0.562 mm. Three fluidic pockets with center-to-center distance of *v*_1_ = 4 mm are drilled on the bottom plane of the HM-1 cavity with the location of first fluidic pocket being at *d*_*x*1_ = 10 mm, *d*_*y*1_ = 10 mm. Similarly, two fluidic pockets with center-to-center distance of *v*_2_ = 4 mm are drilled on the bottom plane of the HM-2 cavity. The location of first fluidic pocket is at *d*_*x*2_ = 8.5 mm, *d*_*y*2_ = 10 mm. For example, the suggested antenna (Ant-4) is poured with distilled water, and the EM-predicted *S*-parameters are shown in Fig. 4d. The operating frequencies are shifted towards lower values of 2.74 GHz and 4.54 GHz which corroborates frequency tunability and further size reduction.

**Fig. 5:**
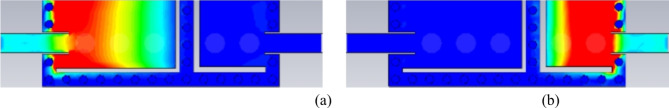
Surface current density at various port excitation: (**a**) Port-1 and (**b**) Port-2.

The self-diplexing property and radiation mechanism are explained by studying the surface-current densities generated by employing excitation at one port and matched termination at the other port and vice-versa. Figure 5 shows the surface-current densities at both ports. When Port-1 is powered, the maximum current flows in the HM cavity associated with Port-1, and almost no current flows to Port-2 as depicted in Fig. 5a. Thus, the radiation occurs through the slot associated with Port-1. Similarly, when Port-2 is powered, the maximum current flows to the HM cavity corresponding to Port-2, and almost zero current flows to Port-1 as seen in Fig. 5b. Hence, the radiation is related to the slot associated with Port-2, which establishes self-diplexing property.

### Circuit model of the antenna without microfluidic pockets

**Fig. 6:**
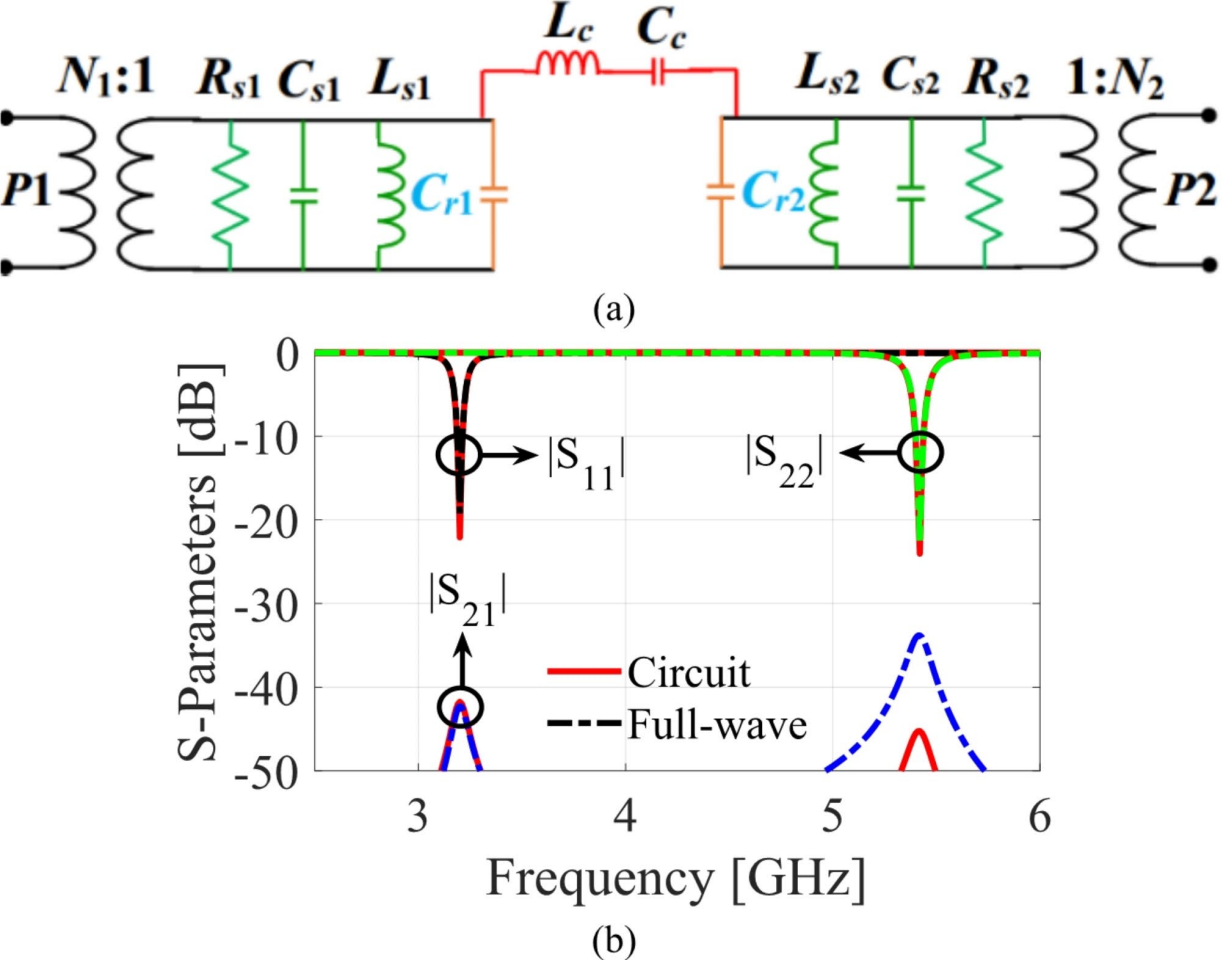
Equivalent circuit model without microfluidic channels: (**a**) circuit diagram, (**b**) circuit and full-wave simulation *S*-parameters.

The suggested antenna diplexer and its performance is validated be creating an equivalent circuit model by following^9–11^. Figure 6a shows circuit model of the antenna diplexer without microfluidic pockets. The parallel connected resistance, inductance, and capacitance are used to model each HM cavity. The first HM cavity is modelled as a shunt connected *R*_*s*1_, *C*_*s*1_, and *L*_*s*1_, whereas the parallel connected *R*_*s*2_, *C*_*s*2_, and *L*_*s*2_ is used to represent the second HM cavity. The LSS loaded on the first and second HM cavities are represented by using *C*_*r*1_ and *C*_*r*2_, respectively. The matching between cavities and the microstrip-lines is obtained using impedance transformers, which are expressed as *N*_1_ and *N*_2_. The electrical parameters are computed as: *R*_*s*1_ = 478 Ω, *C*_*s*1_ = 0.0997 pF, *L*_*s*1_ = 0.3221 nH, *C*_*r*1_ = 7.6796 pF, *R*_*s*2_ = 371 Ω, *C*_*s*2_ = 0.04 pF, *L*_*s*2_ = 0.124 nH, *C*_*r*2_ = 6.9537 pF, *L*_*c*_ = 25.50 nH, *C*_*c*_ = 4.4 pF, *N*_1_ = 0.229, *N*_2_ = 0.349. Table 1 summarizes the relationship between the ECM parameters and the antenna diplexer’s physical dimensions. Based on the electrical parameters, the proposed circuit model is analyzed using Keysight ADS tool and the computed results are compared with the EM-predicted *S*-parameters as depicted in Fig. 6b. It is observed that the circuit and EM-predicted *S*-parameters are well-aligned.

**Table 1:** The dimensions of the proposed antenna diplexer are related to the equivalent circuit model.

Equivalent Circuit Model	Proposed antenna diplexer model
Resistors (*R*_*s*1_ and *R*_*s*2_)	Width and length of the cavities (*W*_*p*1_, *W*_*p*3_, and *W*_*p*4_) and placement of the excitation ports
Capacitors (*C*_*s*1_ and *C*_*s*2_)	Width and length of the cavities (*W*_*p*1_, *W*_*p*3_, and *W*_*p*4_)
Inductors (*L*_*s*1_ and *L*_*s*2_)	Width and length of the cavities (*W*_*p*1_, *W*_*p*3_, and *W*_*p*4_)
Capacitors (*C*_*r*1_ and *C*_*r*2_)	Width and length of the slots (*L*_*s*1_, *L*_*s*2_, *L*_*s*3_, *L*_*s*4_, *W*_*s*1_, and *W*_*s*2_)
Impedance transformer ratios (*N*_1_ and *N*_2_)	Placement of the ports and dimensions of the feed lines

### Circuit model of the antenna with microfluidic pockets

**Fig. 7:**
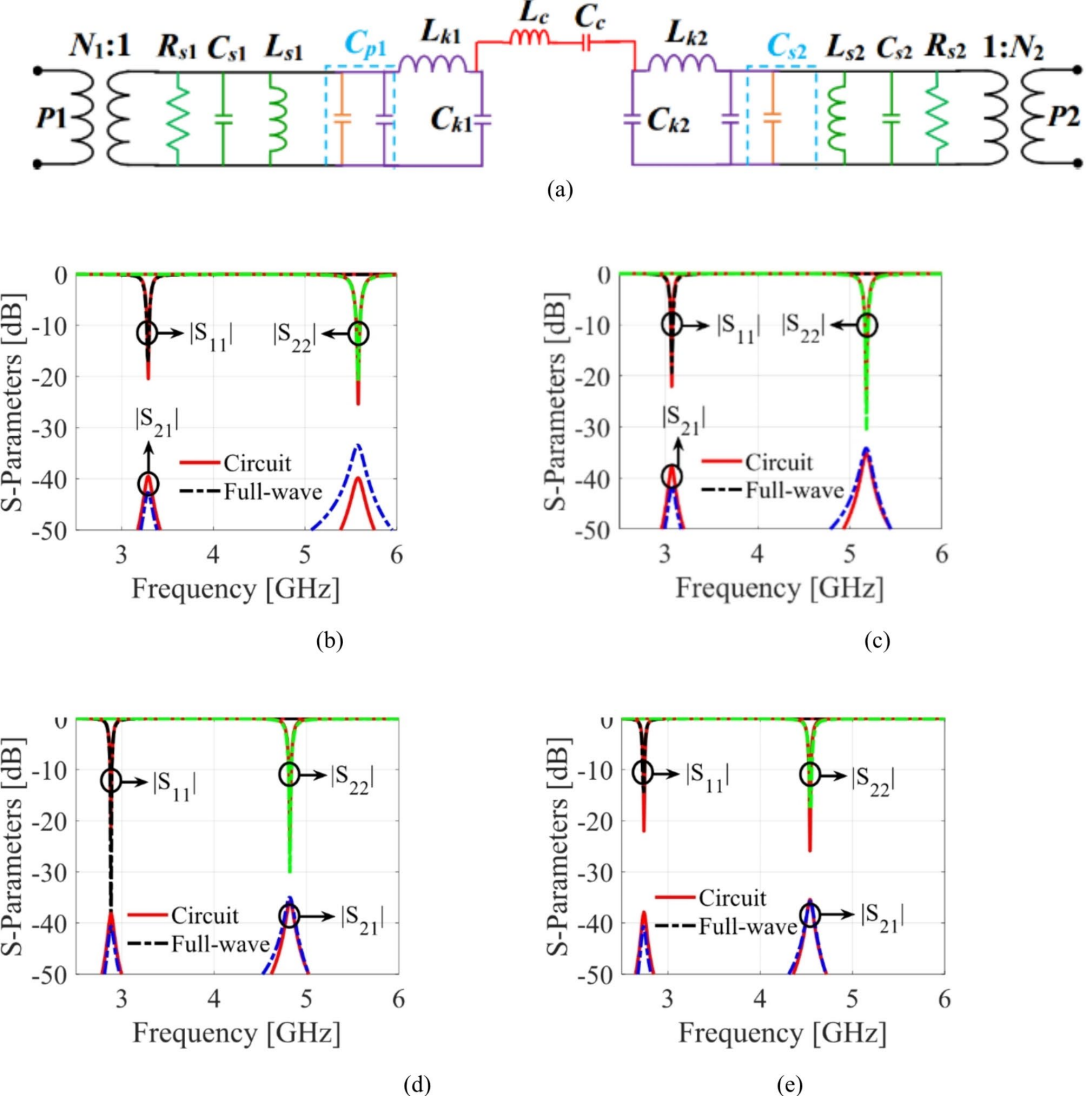
Equivalent circuit model with microfluidic channels: (**a**) circuit diagram, circuit and full-wave simulation *S*-parameters for various loadings: (**b**) air, (**c**) ethyl acetate, (**d**) acetone, and (**e**) distilled water.

An equivalent circuit is also created to validate the suggested antenna diplexer loaded with microfluidic pockets, as depicted in Fig. 7a. The explanation of the circuit model holds the same as that of Fig. 6a, with the additions related to the fluidic pockets. A П-network with a series inductance (*L*_*k*1_/*L*_*k*2_) connected between two shunt capacitances (*C*_*k*1_/*C*_*k*2_) represents the fluidic channels assuming no significant loss by following^21^. The capacitance *C*_*p*1_/*C*_*p*2_ is expressed as the parallel combination of *C*_*r*1_/*C*_*r*2_ and *C*_*k*1_/*C*_*k*2_. The electrical parameters for various dielectric fluids are listed in Table 2, whereas other parameters remain the same as in the case of without microfluidic channels. The circuit-simulated *S*-parameters for air, ethyl acetate, acetone, and distilled water are compared with that of the EM predicted *S*-parameters as depicted in Fig. 7b,c,d,e, respectively. The EM- and circuit model-evaluated *S*-parameters are in good alignment as predicted.

**Table 2:** Parameter values of the circuit model.

Materials	Electrical parameters
Air	*C*_*p*1_ = 4.5907 pF, *Lk*_1_ = 0.2498 nH, *Ck*_1_ = 2.1189 pF, *C*_*p*2_ = 4.4007 pF, *Lk*_1_ = 0.0452 nH, *Ck*_1_ = 1.931 pF
Ethyl acetate	*C*_*p*1_ = 4.8107 pF, *Lk*_1_ = 0.3998 nH, *Ck*_1_ = 2.3789 pF, *C*_*p*2_ = 4.4007 pF, *Lk*_1_ = 0.1052 nH, *Ck*_1_ = 2.381 pF
Acetone	*C*_*p*1_ = 5.8407 pF, *Lk*_1_ = 0.3598 nH, *Ck*_1_ = 2.6189 pF, *C*_*p*2_ = 4.8407 pF, *Lk*_1_ = 0.1052 nH, *Ck*_1_ = 2.881 pF
Distilled water	*C*_*p*1_ = 6.1807 pF, *Lk*_1_ = 0.3498 nH, *Ck*_1_ = 3.0589 pF, *C*_*p*2_ = 5.1507 pF, *Lk*_1_ = 0.1152 nH, *Ck*_1_ = 3.321 pF

### Effect of the height fluidic packets

**Fig. 8:**
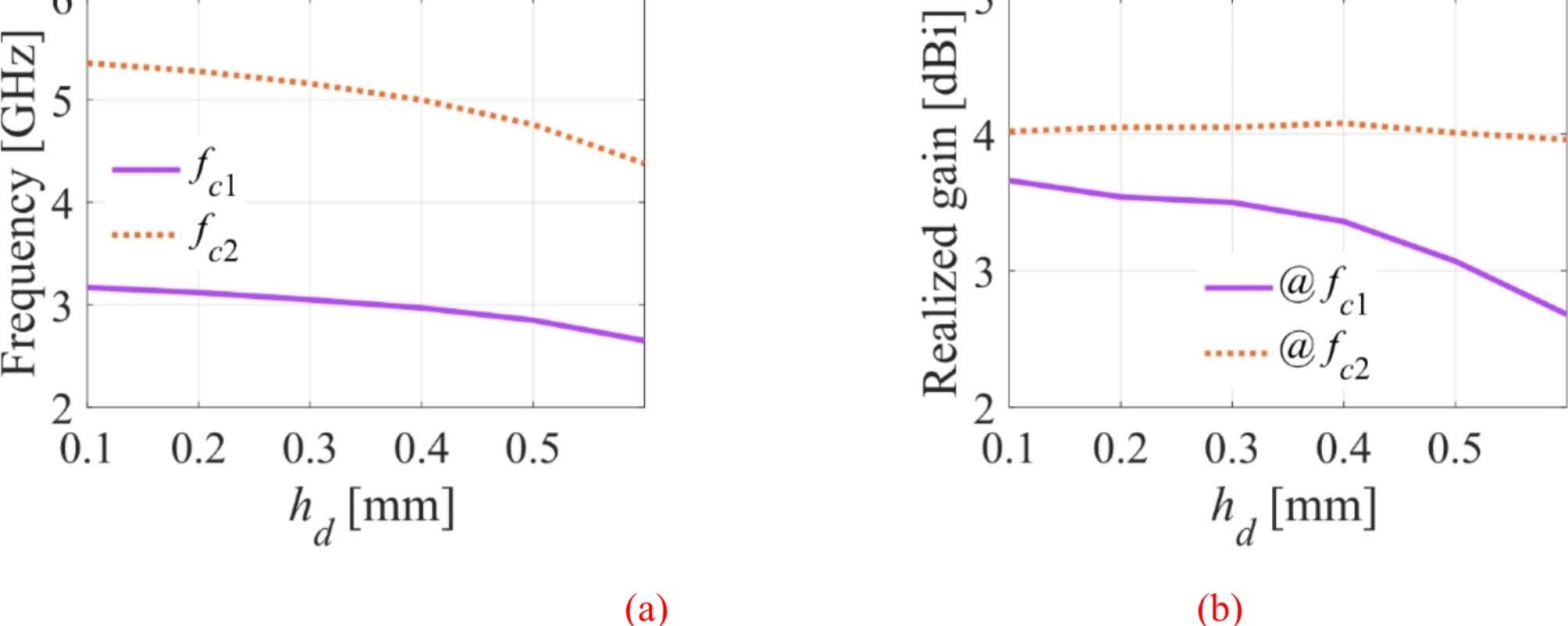
The effects of the height of the fluidic pockets on frequency bands and realized gain: (**a**) variation of frequency bands, (**b**) variation of realized gain.

Assuming that all dimensions of the antenna structure for water-filled pockets remain intact, the effects of the height of the fluidic pockets on the frequency bands and the realized gains are analyzed and shown in Fig. 8. It is observed that the operating frequency bands are shifted towards lower value by increasing the height of the fluidic pockets. Referring to Fig. 8b, note that the realized gain at the upper band remains almost unaltered, but at the lower band it decreases as the height of the pockets increases. The water-filled pockets have very high dielectric permittivity that modifies the total permittivity of the substrate, which results in moving the operating bands to a lower value. It also has a significant effect on the realized gain, which has been traded for to achieve a good tuning range.

### Effect of position of the fluidic packets

**Fig. 9:**
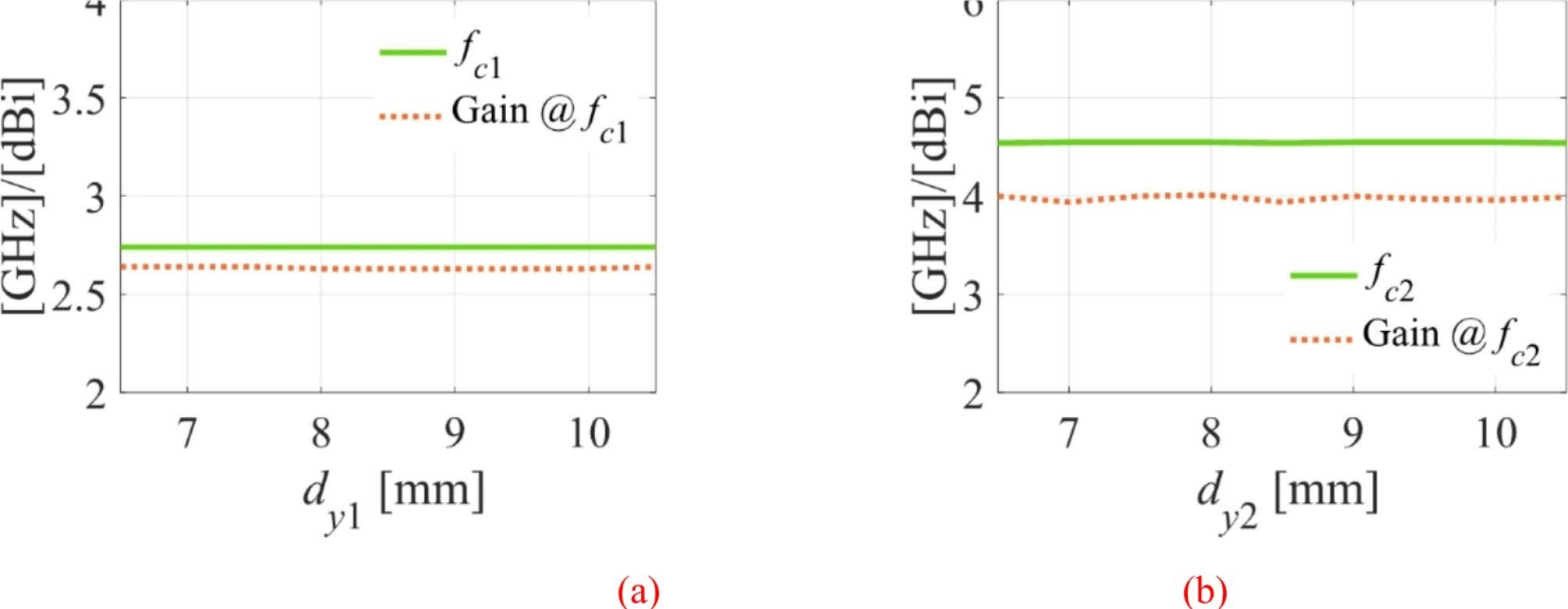
Effect of position of microfluidic pockets on frequency bands and realized gain: (**a**) on HM-1 cavity and (**b**) on HM-2 cavity.

To accomplish frequency reconfigurability, three fluidic packets are implemented for the first HM cavity and two are milled on the ground plane of the second HM cavity. The packets’ specified height and diameter are 2.4 and 0.562 mm, respectively. To prevent any disruption of the radiating aperture, the packets were made smaller than the substrate’s height (0.762 mm) and the substrate’s thickness was left out from the top plane by 0.2 mm. The fluidic packets in this work may be arranged in a single row, according to the EM simulation; otherwise, an impedance mismatch will result. Therefore, a maximum of three packets may be created on the first HM cavity and two on the second HM cavity with the specified cavity dimensions, fluidic packet diameter, and perfect milling of the empty vias. The performance is unaffected by the vertical placements of these rows of fluidic packets. This is explained by examining the impact of the fluidic packets’ vertical positioning on the frequency bands and realized gains, as shown in Fig. 9. It is evident from the figure that the frequency bands and realized gains are not significantly impacted by the fluidic positioning on vertical locations.

### Frequency tunability based on the L-shaped slot

**Fig. 10:**
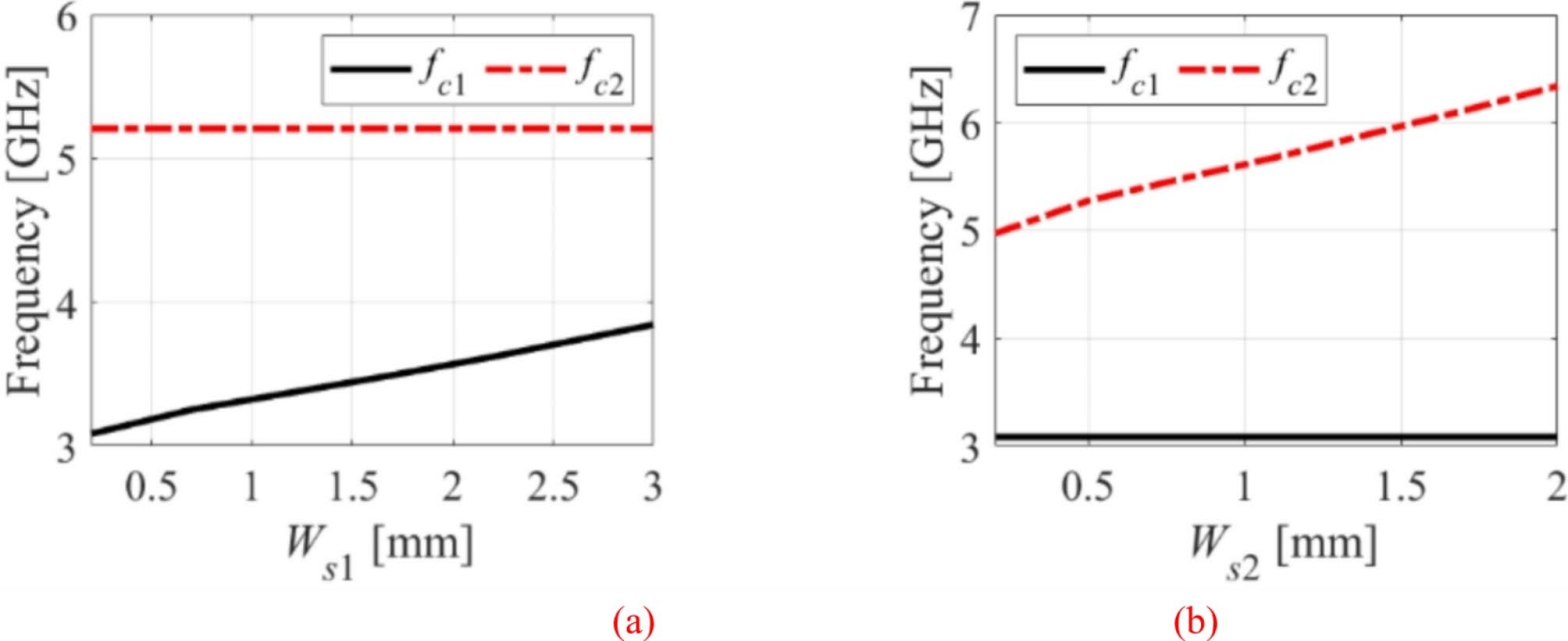
Frequency tunability of the suggested antenna without microfluidic pockets: (**a**) lower band, (**b**) upper band.

In this method, the operating frequencies can be tuned individually or simultaneously by varying the widths of the L-shaped slots. The dimensions *W*_*s*1_ and *W*_*s*2_ of the slots are altered to tune the frequency bands, as shown in Fig. 10. When *W*_*s*1_ is varied from 0.2 to 3.0 mm, the first frequency band alters from 3.08 to 3.84 GHz with a tuning range of 24.6% as shown in Fig. 10a. Similarly, the second frequency band is changed from 4.97 to 6.33 GHz with a tuning range of 27.5% when the parameter *W*_*s*2_ varies from 0.2 to 2 mm, as depicted in Fig. 10b. This approach requires a change in physical dimensions and a new prototype fabrication to attain the appropriate operating frequency, which must be repeated for any further frequency bands. As a result, this approach is unsuited for frequency-agile RF front-end systems.

### Microfluidically-based frequency reconfigurability

**Fig. 11:**
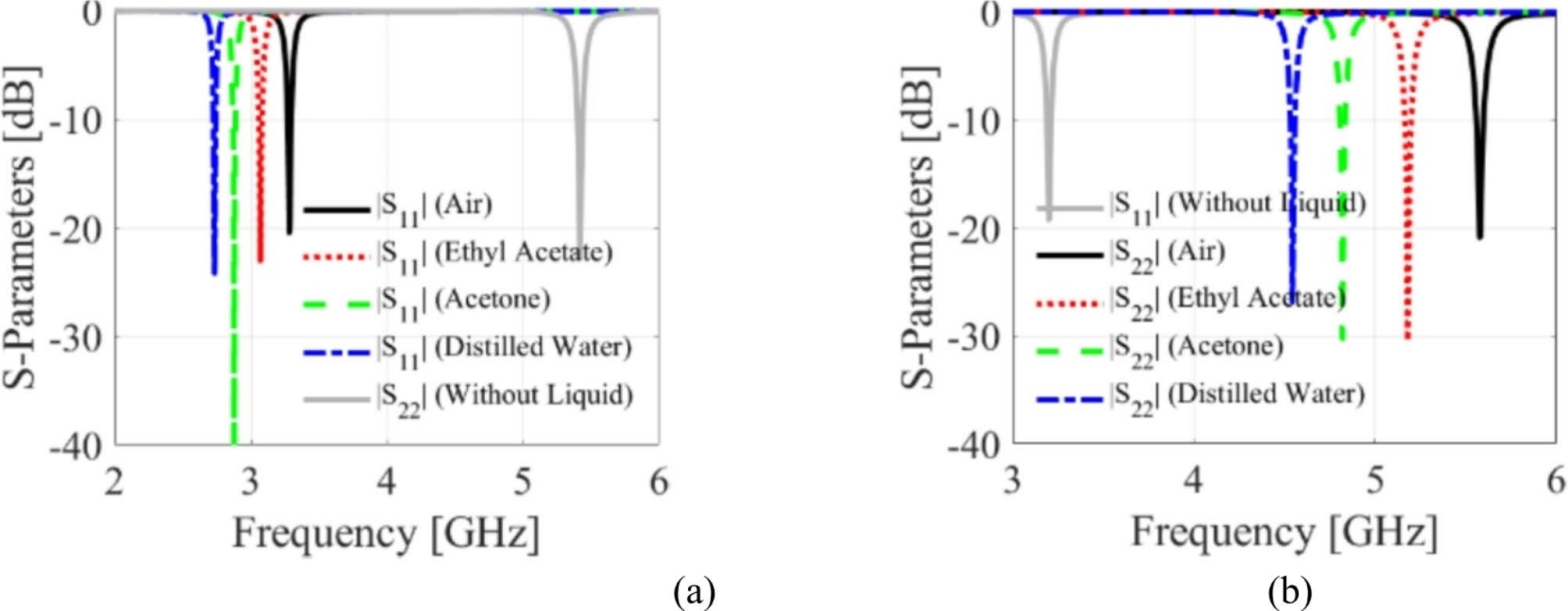
Frequency tunability of the suggested antenna with microfluidic pockets: (**a**) lower band, (**b**) upper band.

 In the light of the above limitation, a microfluidically based tuning method to achieve flexible frequency reconfigurability is analyzed in this section. Three and two empty fluidic pockets are drilled on the bottom conductor of the first and second HM cavities, respectively. The diameter and height of the fluidic pockets are expressed by *d*_1_ = *d*_2_ = 2.4 mm and *h*_*d*_ = 0.562 mm. The fluidic packets are made smaller in height than the substrate (*h*_*s*_ = 0.762 mm), and 0.2-mm-thick substrate substrate layer is left from the top plane to safeguard the radiating apertures. These pockets are poured with air, ethyl acetate, acetone, and distilled water with primitivities of 1, 6.02, 20.7, and 78.7, respectively, to enable reconfigurability. Figure 11 shows the tuning of both frequency bands. Figure 11a shows that the first band is reconfigured from 2.74 GHz to 3.28 GHz with a tuning range of 19.7%, while the second band varies from 4.54 GHz to 5.58 GHz with a tuning range of 22.7% as depicted in Fig. 11b. Therefore, based on the two methods, the first and second frequency bands’ overall tuning ranges are 2.74–3.84 GHz (40.1%) and 4.54–6.33 GHz (39.6%), respectively. Moreover, the proposed self-diplexing antenna offers narrow bandwidth at the operating bands that is suitable for narrowband channel selection of different communication standards. Table 3 enlists possible applications of the suggested antenna diplexer. The communication standards 5G (sub 6-GHz), WLAN, WiFi, and WiMAX consists of several channels with different bandwidths (10 MHz, 20MGz, and 40 MHz). Let us consider WLAN (IEEE 802.11j) which have different channels at 4.91–4.93 GHz, 4.93–4.95 GHz, 4.95–4.97 GHz, 4.97–4.99 GHz, 4.945–4.965 GHz, and so on. These channels can be achieved by using the proposed frequency reconfigurable self-diplexing antenna with high isolation between the channels.

**Table 3:** Application feasibility of the suggested reconfigurable antenna diplexer.

Tuning method	Frequency bands	Tuning range (GHz)	Application
Altering slot widths	Lower-band	3.08–3.84	5G/WiMAX/WLAN
	Upper-band	4.97–6.33	WLAN/WiFi/WMAN/WiMAX
Reconfigurability based on fluidic pockets	Lower-band	2.74–3.38	5G/WiMAX/LTE
	Upper-band	4.54–5.58	5G/WLAN/WiFi/WMAN

## Results

### Fabrication and measurement

**Fig. 12:**
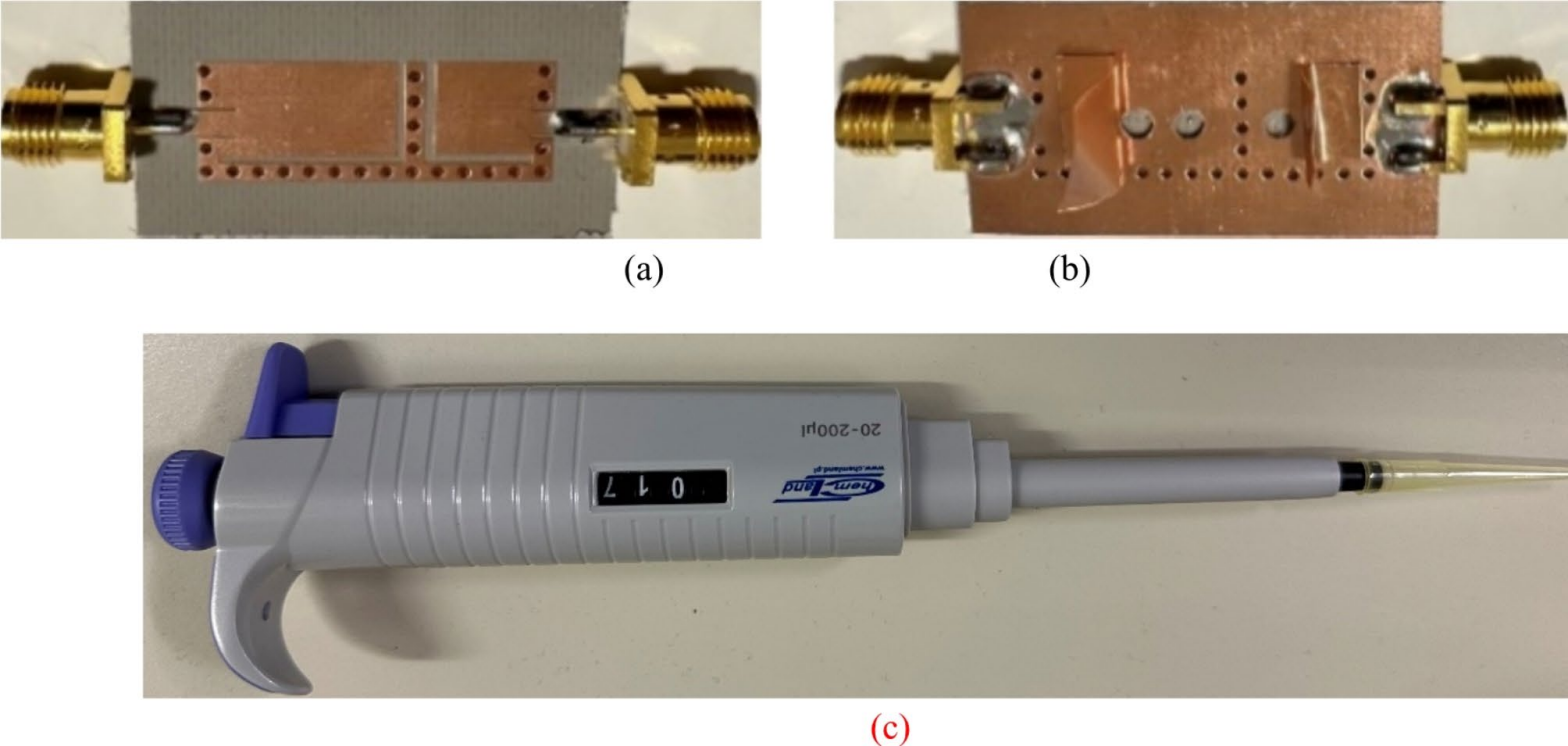
Photographs of the fabricated antenna diplexer: (**a**) front view, (**b**) back view, and (**c**) volume-controlled fluid injector/retractor.

**Fig. 13:**
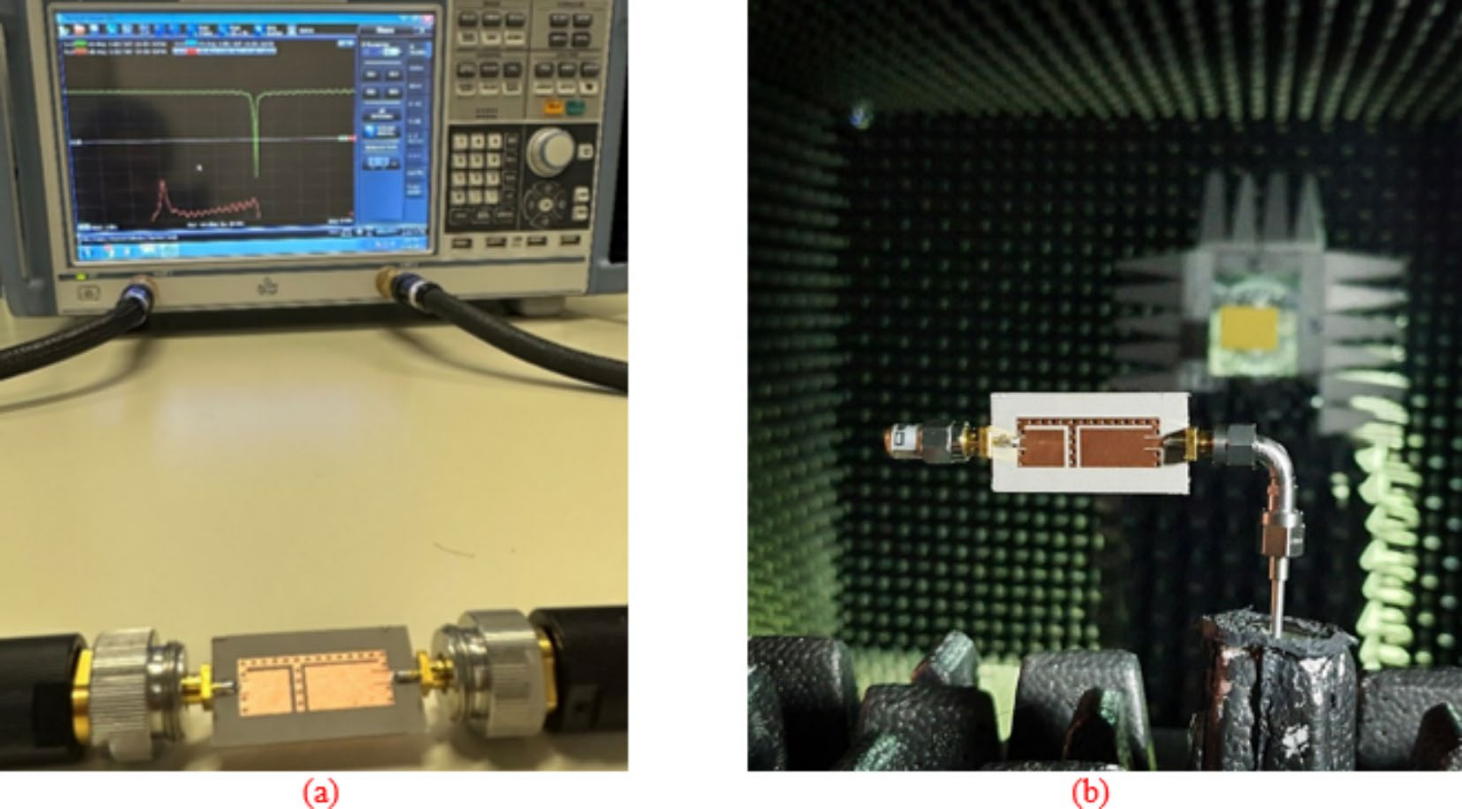
Measurement setup for the suggested antenna diplexer: (**a**) S-parameters, and (**b**) far-field performance.

**Fig. 14:**
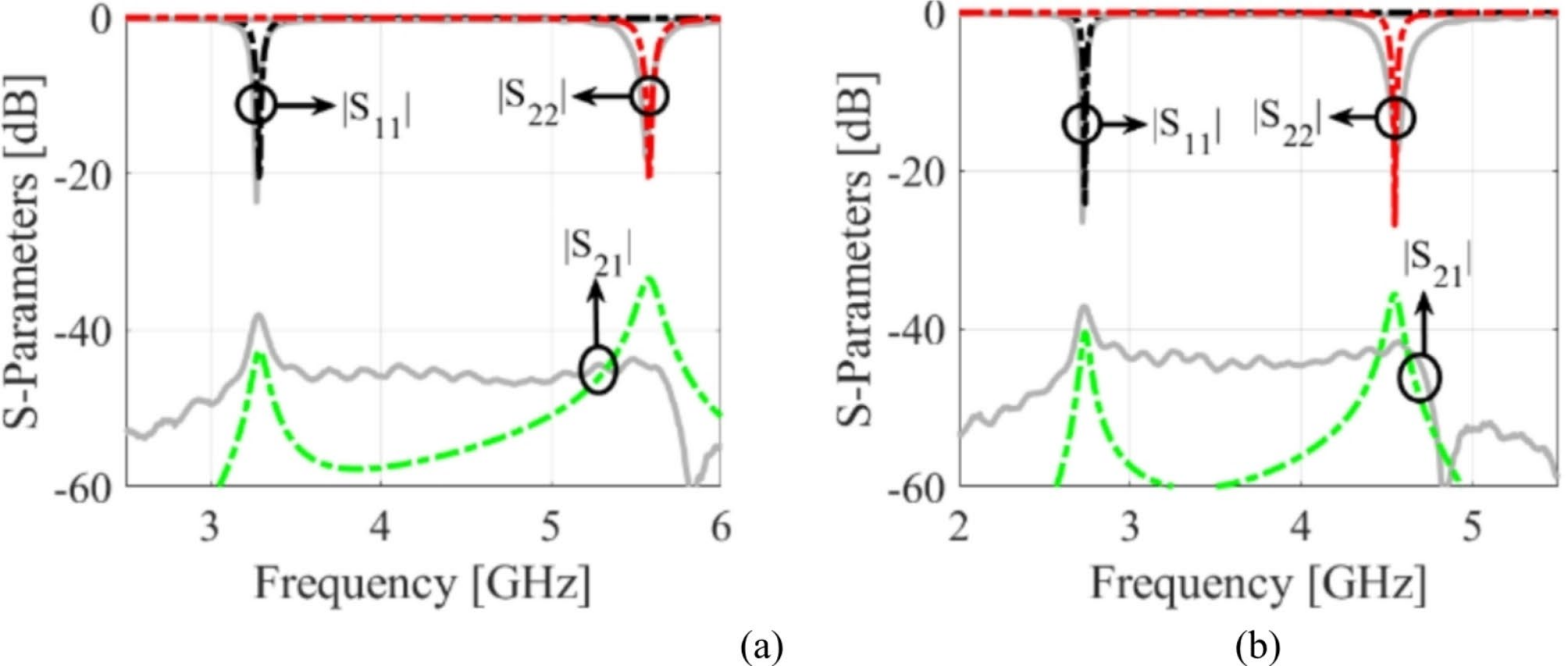
Measured (solid) and EM predicted (dotted) S-parameters for: (**a**) Air-filled pockets and (**b**) water-filled pockets.

A prototype of the suggested antenna diplexer is fabricated on an 0.762-mm thick Roger’s AD250 substrate (*ε*_*r*_ = 2.5 and tan*δ* = 0.0014). Figure 12 depicts the front and rear views of a constructed antenna, as well as a volume controlled fluidic injector and retractor. To ease reconfigurability, a single-channel volume-controlled pipette is used to inject/retract fluid into the channels with the appropriate volume. After the channels are filled, copper tape is applied to the channels to restore the resonator’s ground plane, as seen in Fig. 12b. Similarly, the pipette retracts the fluid by creating a vacuum and drawing fluid into its chamber. Consequently, various dielectric liquids may be used to attain the desired operating frequencies. Figure 11 depicts the measuring setup for the manufactured antenna to exhibit S-parameters and radiation characteristics. The S-parameter measurement was carried out using the Rohde and Schwarz vector network analyzer, as shown in Fig. 13a. As shown in Fig. 13b, an automated anechoic chamber was employed to capture the realized gains and normalized radiation patterns. The *S*-parameters of the antenna are measured by filling air and water into the empty vias as depicted in Fig. 14. Referring to Fig. 14a, it is observed that the input return losses and isolation for air-filled vias exceed − 20.3 and 33.4 dB. Similarly, the input return losses and isolation for water-filled vias are better than − 21.3 and 33.3 dB, as depicted in Fig. 14b. The measurement (EM simulation) fractional 10-dB matching bandwidths for air-filled pockets at the lower and upper frequency bands are 30 MHz (36 MHz) and 40 MHz (45 MHz), respectively. Similarly, the measurement (EM simulation) fractional 10-dB matching bandwidths for water-filled pockets at the lower and upper frequency bands are 24 MHz (29 MHz) and 28 MHz (75 MHz), respectively. Due to implementing a practically important feature of independent tuning, we have achieved a narrow bandwidth in two application bands. This allows us to use for pinpoint applications in the mentioned commercial wireless application bands, such as WLAN, WiMAX, WiFi, and 5G, where the required individual channel bandwidths are 10, 20, and 40 MHz. However, the bandwidths can be improve by employing various techniques such as increasing substrate thickness, increasing loss, using slots, and dielectric resonator antennas^22,23^.

### Far-field performances

**Fig. 15:**
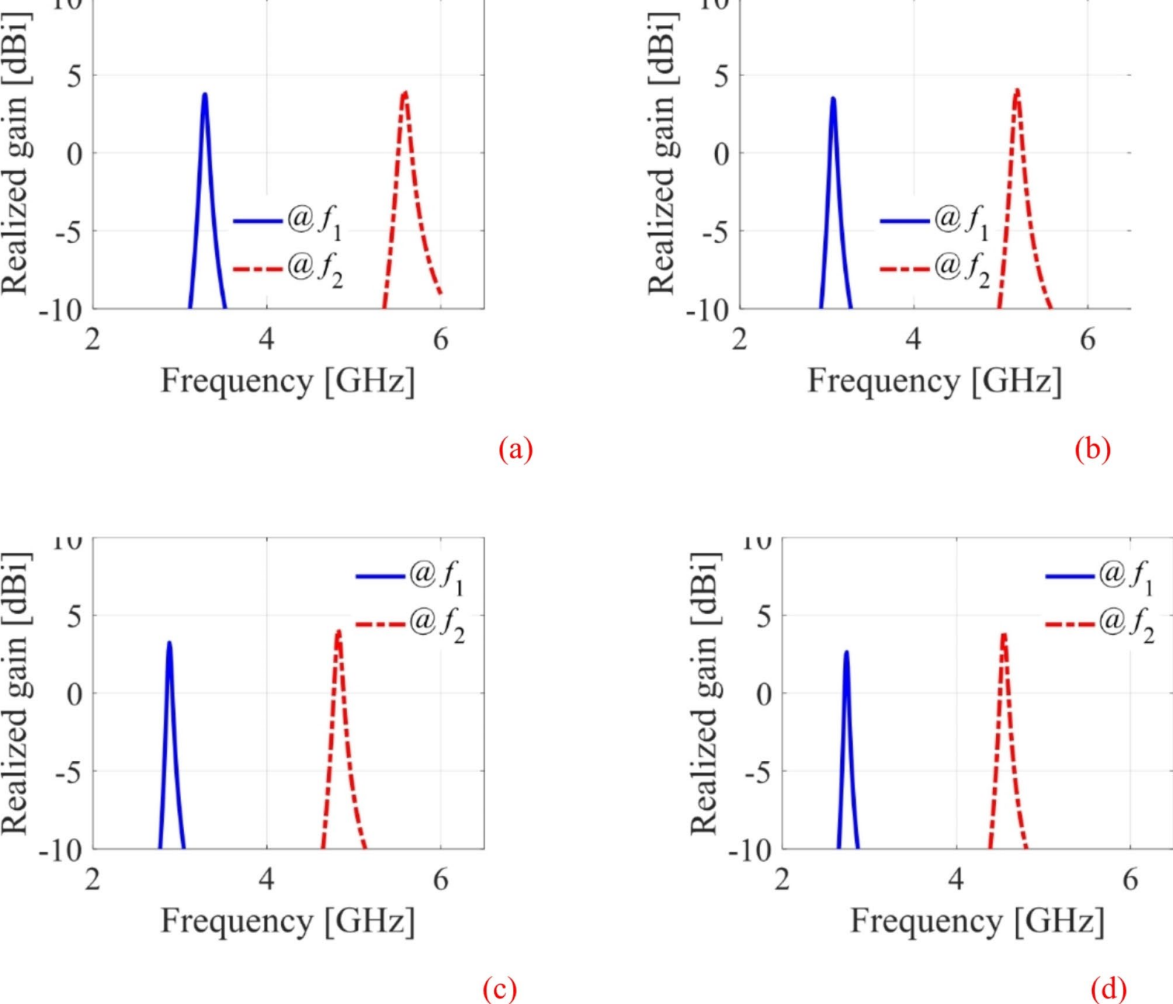
EM Simulated realized gains for different dielectric fluids. (**a**) air-filled pockets, and (**b**) ethyl acetate-filled pockets, (**c**) acetone-filled pockets, (**d**) water-filled pockets.

**Fig. 16:**
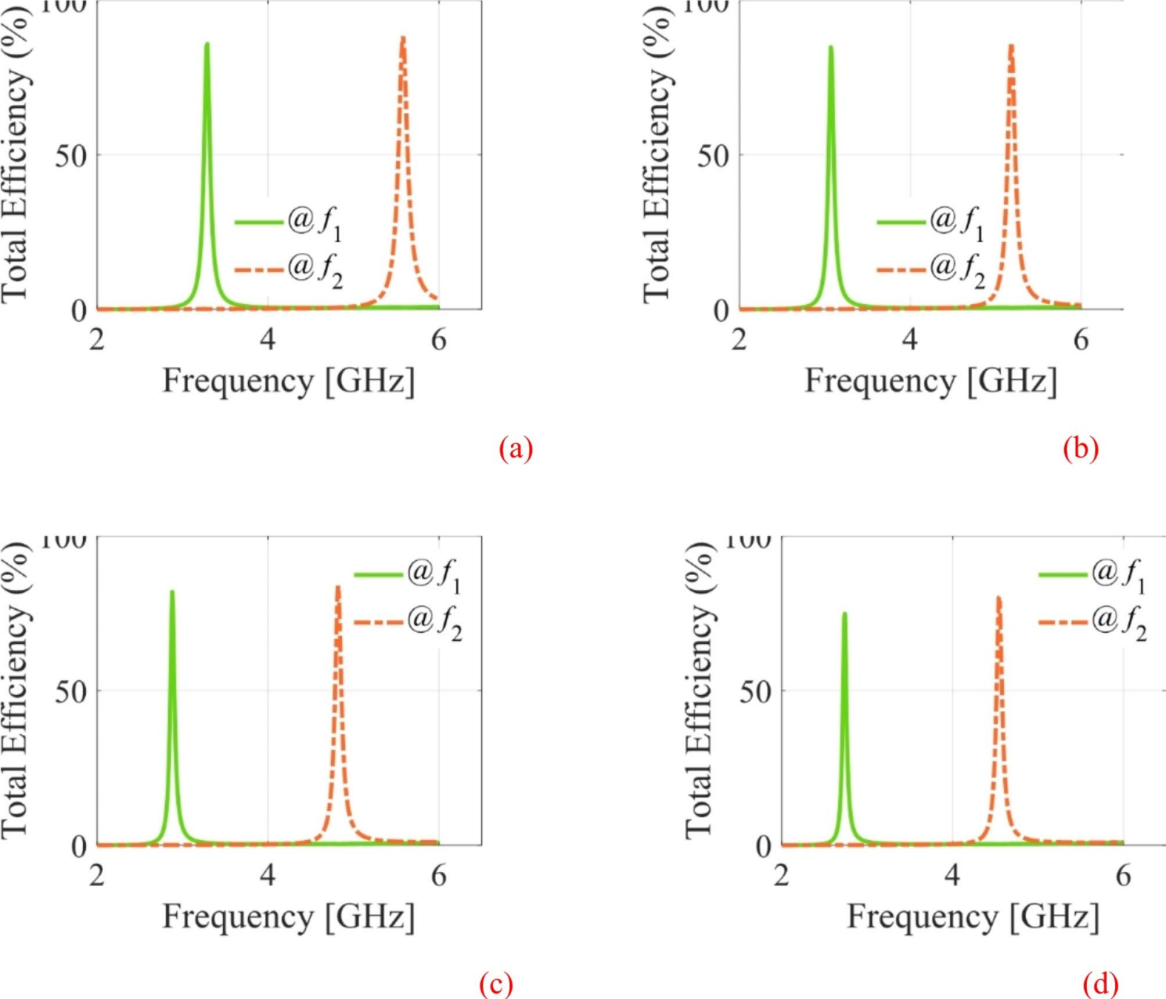
EM Simulated total efficiency for different dielectric fluids. (**a**) air-filled pockets, and (**b**) ethyl acetate-filled pockets, (**c**) acetone-filled pockets, (**d**) water-filled pockets.

**Fig. 17:**
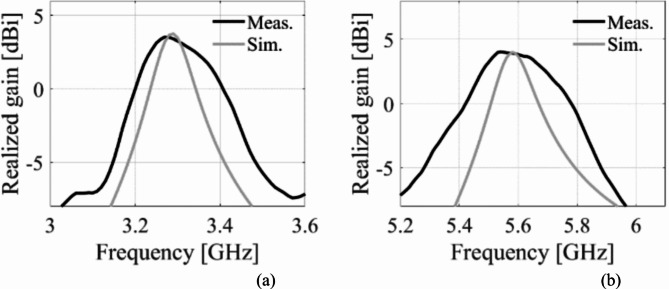
Measured and EM predicted realized gain for air-filled pockets. (**a**) at 2.29 GHz, and (**b**) at 5.08 GHz.

**Fig. 18:**
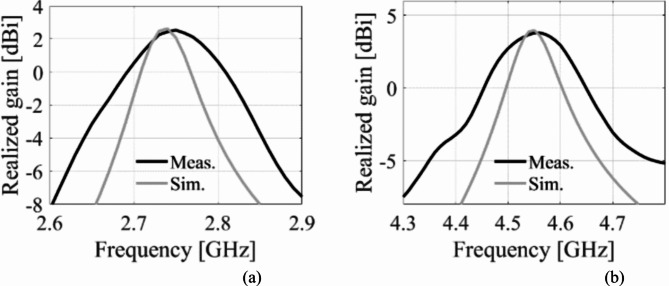
Measured and EM predicted realized gain for water-filled pockets. (**a**) at 2.29 GHz, and (**b**) at 5.08 GHz.

**Fig. 19:**
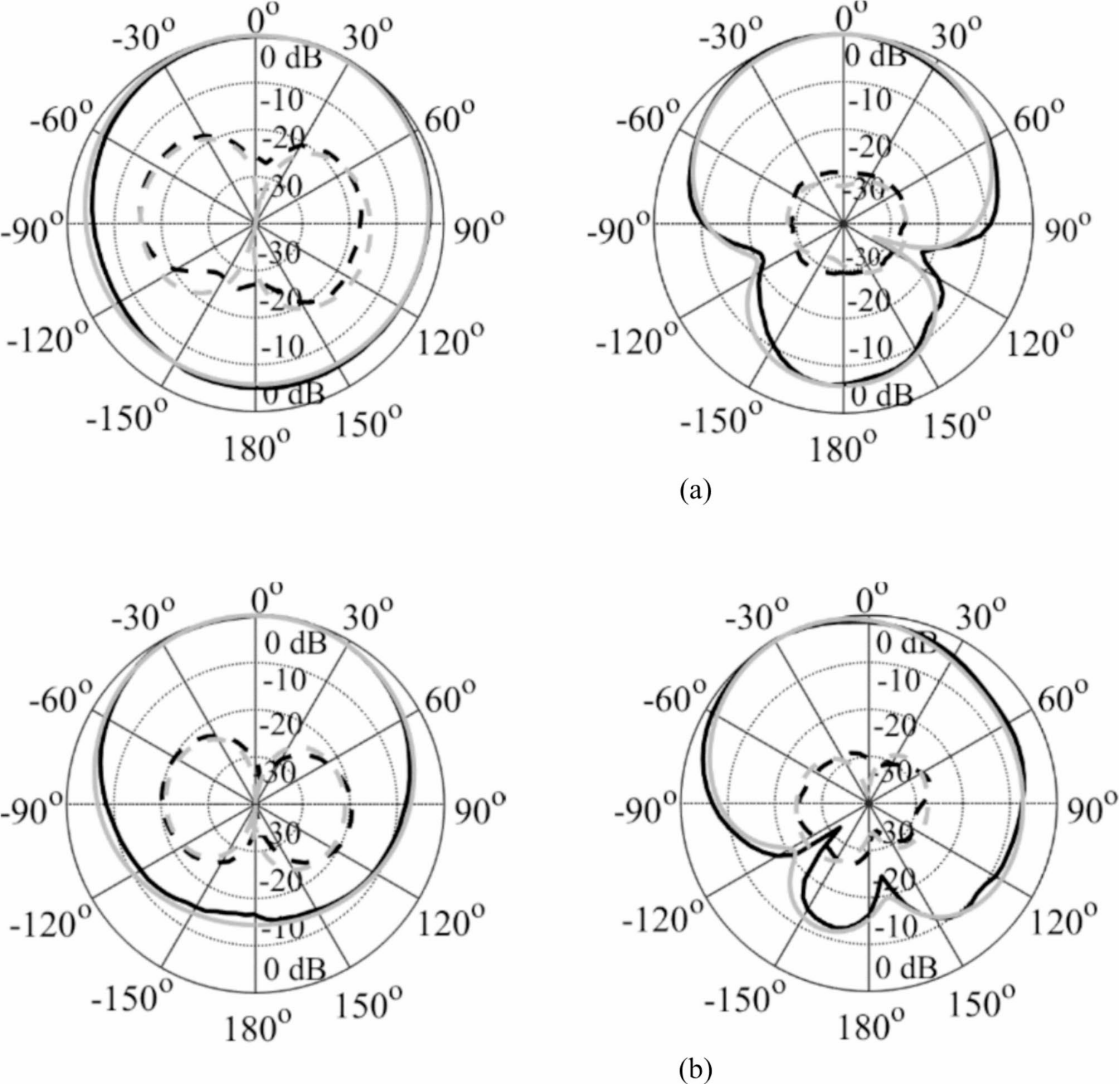
Simulated (grey) and measured (black) normalized radiation patterns for water-filled pockets. (**a**) at 3.28 GHz and (**b**) at 5.58 GHz. [H-plane (left), E-plane (right); copol – solid, crosspol – dashed].

**Fig. 20:**
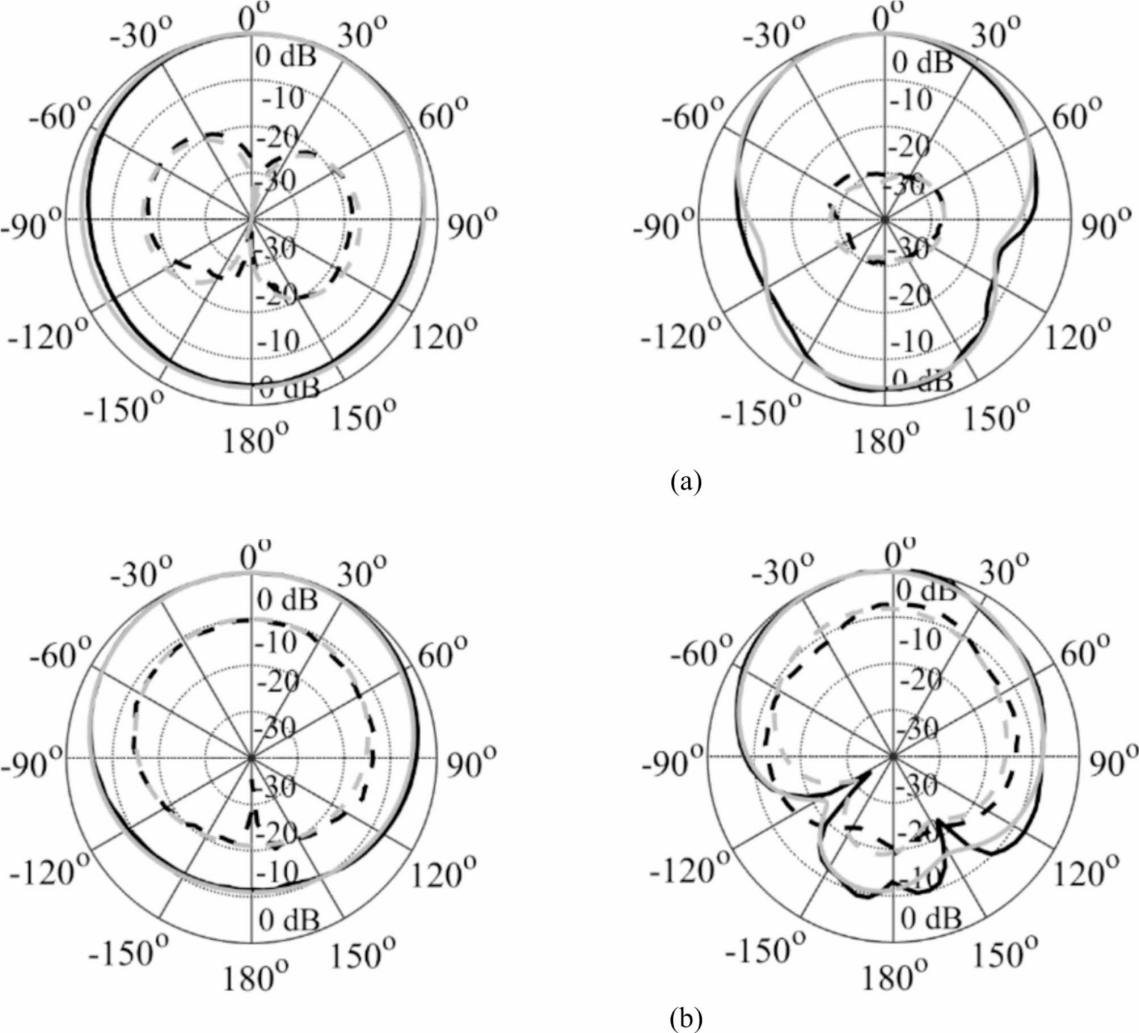
Simulated (grey) and measured (black) normalized radiation patterns for water-filled pockets. (**a**) at 2.74 GHz and (**b**) at 4.54 GHz. [H-plane (left), E-plane (right); copol – solid, crosspol – dashed].

The EM simulated realized gain for different dielectric liquids is computed as illustrated in Fig. 15. It is observed that the realized gain at the lower and upper frequency bands is greater than 3dBi and 4dBi, respectively. The EM simulated total efficiency has been analyzed for various dielectric liquids, as shown in Fig. 16. It is observed that the total efficiency at the lower and the upper frequency bands are greater than 75% and 80% for all four cases. However, the far-field radiation properties of the fabricated antenna diplexer are measured using an automatic anechoic chamber for air- and water-filled packets only. The realized gains and radiation patterns are determined by applying excitation at Port-1 and terminated matching at Port-2 and vice-versa. The measured EM predicted realized gains for air- and water-filled vias are depicted in Figs. 17, 18, respectively. The measured (EM predicted) realized gains at the broadside for air-filled pockets are 2.51dB (2.62dB) and 3.82dB (3.94dB) at 2.74 and 4.54 GHz, respectively. Similarly, for water-filled pockets, the measured (EM predicted) realized gains at the broadside for air-filled pockets are 3.54dB (3.75dB) and 3.82dB (3.98dB) at 3.28 and 5.58 GHz, respectively. The radiation patterns of the proposed antenna diplexer with air- and water-filled pockets are illustrated in Figs. 19, 20, respectively. It is seen that the suggested antenna suffers from the squint problem in the E-plane cut at high-band due to the input feeding lines’ poor matching. To obtain appropriate matching for different fluidic pockets, the inline feedings’ dimensions are carefully designed. As a result, although the suggested antenna construction provides excellent matching for all fluidic pockets, it does not provide the optimum matching for any of them. As a result, the suggested high-band antenna has squint problems in the E-plane cut. From the Figures, it is observed that the antenna prototype offers linear polarization and a unidirectional pattern. The EM-predicted and measured results are well-aligned. However, a small discrepancy is observed due to fabrication tolerance, SMA connector loss, and imperfect dielectric material.

The performance of the proposed design is compared with the previously reported tunable self-diplexing antennas as highlighted in Table 4. The designs in^6–11^ report self-diplexing antennas with fixed operating bands. Therefore, for a complete comparative analysis among the self-diplexing antennas, we have included the designs reported in^6–11^ whose operating bands are fixed, and the tunable self-diplexing antennas in^13–15,18^. It can be observed that the circuit area of the proposed design is 81.2, 89.9, 84.6, and 79.5% smaller than the antennas reported in^13–15,18^, respectively. It is also found that the isolation of the proposed design is highest among the existing tunable antennas^13–15,18^. Additionally, the proposed design offers broader frequency tuning ranges at different operating bands. Since this method of frequency reconfigurable is entirely passive and discrete, the use of dielectric liquids in an auto-fill technique for frequency adjusted self-diplexing antennas will be explored.

**Table 4:** Comparison with state-of-the-arts self-diplexing antennas.

Ref.	Operating bands	Freq. bands (GHz)	ISL (dB)	Realized Gain (dBi)	Size (λ_g_^2^)	Circuit model
^6^	Fixed	0.92/1.85	> 17	1.3/4.4	0.74	No
^7^	Fixed	6.62/11.18	> 29.3	5.42/5.66	0.462	No
^8^	Fixed	4.29/7.52	> 32.8	5.38/5.82	0.23	No
^9^	Fixed	3.47/3.84	> 47	4.8/23.5	0.15	Yes
^10^	Fixed	3.6/5.4	> 32.5	4.9/5.34	0.08	Yes
^11^	Fixed	3.5/4	> 23	10/11.5	7.84	Yes
^13^	Tunable	3.88–4.37 4.61–5.33	> 22	0.8/0.9	0.24	Yes
^14^	Tunable	2.64–2.82 3.15–3.41	> 20	4.6/4.9	0.448	Yes
^15^	Tunable	3.77–4.59 4.96–6.5	> 21	4.85/4.96	0.294	Yes
^18^	Tunable	3.5–3.8 5.53–6.2	> 27	5.04/5.26	0.22	Yes
Proposed	Tunable	2.74–3.84/ 4.54–6.33	> 33.4	2.62–3.75/ 3.94–3.92	0.045	Yes

## Conclusion

In this brief, an ultra-miniaturized frequency tunable antenna diplexer based on fluidic packets has been designed. In the first design stage, two half-mode substrate-integrated cavities are connected side-by-side and loaded with L-shaped slots (LSS) on the top conductors. The LSSs are excited by two microstrip feedlines to obtain two fixed operating bands of 3.2 GHz and 5.42 GHz. These frequency bands are tuned by employing two mechanisms: (i) varying the dimensions of the LSSs, and (ii) loading various dielectric liquids into fluidic vias created on the bottom conductor. The suggested antenna diplexer has been validated by analyzing an equivalent circuit model without and with dielectric liquids. Finally, the proposed antenna diplexer prototype has been manufactured and experimentally demonstrated. The fabricated antenna has a highly compact footprint of 0.045λ_g_^2^. It exhibits a high isolation of greater than 33 dB with a realized gain of exceeding 2.62 dBi at both frequency bands. The antenna prototype has a wide frequency tuning range of 2.74–3.84 and 4.54–6.33 GHz at lower and upper operating bands, respectively. The EM-predicted results are well validated through circuit and measurement demonstrations.

## Data Availability

The datasets used and/or analyzed during the current study are available from the corresponding author on reasonable request.
